# Supplementary material for: Systematic Review and Meta-Analysis: Accuracy of Both Gamma Delta+ Intraepithelial Lymphocytes and Coeliac Lymphogram Evaluated by Flow Cytometry for Coeliac Disease Diagnosis
Source: Nutrients. 2019 Aug 23;11(9):1992. doi: 10.3390/nu11091992 (PMC6769802; doi:10.3390/nu11091992)
Supplement: Supplementary file 1 [file nutrients-11-01992-s001.pdf]

Supplementary files:

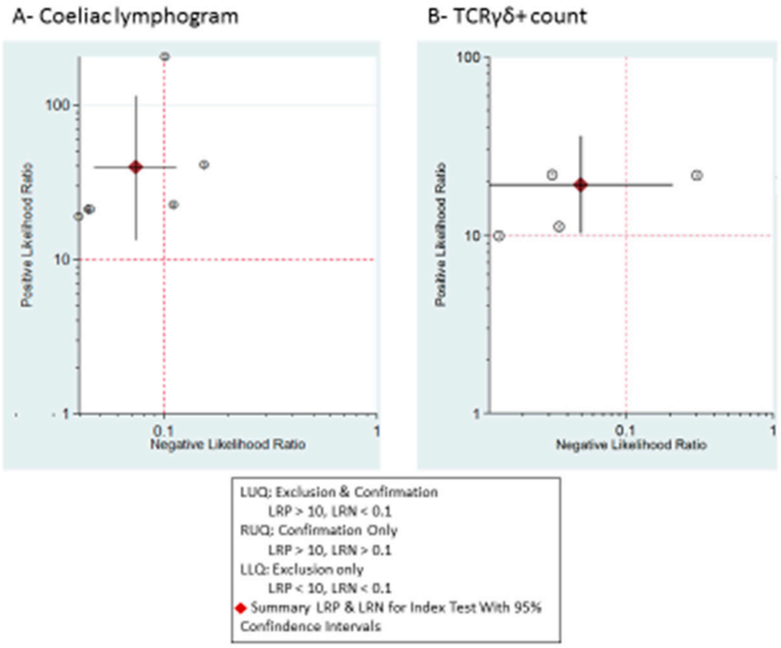

Supplementary Figure S1. Likelihood matrix for the overall distribution of the studies: (**A**) Coeliac lymphogram; (**B**) TCRγδ^{+}. Each point corresponds to a study. The summary positive likelihood ratio (LRP) and negative likelihood ratio (LRN) for index test are found on the left upper quadrant of the matrix, and showed a high accuracy for both exclusion and confirmation of CD.

Supplementary Table S1. Methodological quality of the flow cytometry methods.

MIFlowCyt Checklist	Camarero et al., 2000	Calleja et al., 2011	Fernández-Bañares et al., 2014	Saborido et al., 2018	Valle et al., 2017	Nijeboer et al., 2019
1. Experiment overview						
Purpose, experiment variables, time period	P	P	Y	P	Y	Y
Quality control measures (replicates, isotypes, calibration, etc.)	N	Y	Y	N	Y	Y
2. Sample and specimen details						
Sample specimen description (region, number of biopsies)	P	Y	Y	P	Y	Y
Sample treatment description (IEL isolation protocol)	Y	Y	Y	Y	Y	Y
Fluorescence reagents description	Y	P	Y	P	P	P
3. Instrument details						
Manufacturer, model, configuration	Y	Y	Y	N	Y	Y
4. Data analysis details						
Compensation details, data transformation details	N	N	N	N	N	N
Gating details	Y	Y	Y	Y	Y	Y
5. Data presentation						

Axis legends, graphical example of full gating strategy	N	Y	Y	N	N	Y
Positive or negative controls, FMOs	N	N	N	N	N	N

References: Camarero, C.; et al. Intraepithelial lymphocytes and coeliac disease: Permanent changes in CD3−/CD7+ and T cell receptor γβ subsets studied by flow cytometry. *Acta Paediatr.* **2000**, *89*, 285–290; Calleja, S.; et al. Dynamics of Non-conventional Intraepithelial Lymphocytes—NK, NKT, and γδ T—In Celiac Disease: Relationship with Age, Diet, and Histopathology. *Dig. Dis. Sci.* **2011**, *56*, 2042–2049; Fernandez-Bañares, F.; et al. Intestinal Intraepithelial Lymphocyte Cytometric Pattern Is More Accurate than Subepithelial Deposits of Anti-Tissue Transglutaminase IgA for the Diagnosis of Celiac Disease in Lymphocytic Enteritis. *PLoS ONE* **2014**, *9*, e101249; Saborido, R.; et al. Intraepithelial lymphocyte immunophenotype: A useful tool in the diagnosis of celiac disease. *J. Physiol. Biochem.* **2018**, *74*, 153–158; Valle, J.; et al. Flow cytometry of duodenal intraepithelial lymphocytes improves diagnosis of celiac disease in difficult cases. *United Eur. Gastroenterol. J.* **2017**, *5*, 819–826; Nijeboer, P.; et al. Gamma-Delta T Lymphocytes in the Diagnostic Approach of Coeliac Disease. *J. Clin. Gastroenterol.* **2019**, *53*, e208–e213.
Based on Lee JA, et al. MIFlowCyt: the minimum information about a flow cytometry experiment. Cytometry A 2008;73:926-30. Y: Yes; N: No; P: Partially.
